# Health Consequences of an Armed Conflict in Zamboanga, Philippines Using a Syndromic Surveillance Database

**DOI:** 10.3390/ijerph15122690

**Published:** 2018-11-29

**Authors:** Miguel Antonio Salazar, Ronald Law, Volker Winkler

**Affiliations:** 1 Institute of Global Health, University of Heidelberg, Im Neuenheimer Feld 324, 69120 Heidelberg, Germany; 2 Graduate School, Angeles University Foundation, Angeles City, Pampanga 2009, Philippines; 3 Department of Health, Health Emergency Management Bureau, Sta. Cruz, Manila 1003, Philippines; rplaw@doh.gov.ph; 4 College of Public Health, University of the Philippines, Manila 1000, Philippines

**Keywords:** disasters, armed conflict, complex emergencies, syndromic surveillance

## Abstract

The Zamboanga armed conflict was a 19-day long encounter in the Philippines in 2013 that displaced 119,000 people from their homes. This study describes the health consequences of this complex emergency in different age groups, time periods, and health facilities using data from Surveillance in Post Extreme Emergencies and Disasters (SPEED). This is a descriptive study of the SPEED database spanning 196 days of observation post-disaster and 1065 SPEED reports from 49 health facilities. Evacuation centers and village health centers, both primary care facilities, had the highest number of consults. Common infections and noncommunicable diseases were the most common reasons for consultations, namely, acute respiratory infections, fever, watery diarrhea, skin disease, and hypertension. Infections can be associated with environmental conditions in displaced populations, while hypertension has a high prevalence in the country and implies long-term care. Conflict-related injuries and deaths were not frequently observed due to the volatile situation that influenced health-seeking behavior as well as possible reporting gaps. In conclusion, in complex emergencies, as in natural disasters, wherein early alert and warning for potential outbreaks is crucial, SPEED can assist decision makers on response and recovery interventions. Linkages between SPEED and other surveillance and reporting systems need to be explored.

## 1. Introduction

On September 9, 2013, in the city of Zamboanga, Philippines, a gun battle ensued between the Philippine Armed Forces and the Moro National Liberation Front (MNLF). The MNLF engaged government forces in five villages, or barangays, the country’s smallest governance division. The hostilities ended 19 days after the start of the encounter. During the course and in the aftermath of the military skirmish, 118,819 individuals were affected and evacuated from their homes [1]. Six months after the encounter, only half of the affected population were able to return to their homes. The conflict compromised the nutrition and hygiene of the 20,000 internally displaced people in the two largest evacuation sites [2]. The Zamboanga armed conflict may be considered a complex emergency, as it fits the definition of a humanitarian event due to armed conflict, political destabilization, food supply shortages, displacement of populations, and health systems disruption [3].

Conflict and the resulting displacement of affected residents leads to health consequences. Traditionally, communicable diseases have been the focus of disaster management and refugee health [4,5]. Displaced populations are particularly susceptible to communicable diseases because of risk factors such as cramped housing, environmental damage, economic difficulty, lack of hygiene and clean water supply, inadequate nutrition, and lack of basic health services. Diarrhea and acute respiratory infections are the two most common causes of morbidity and mortality in displaced populations [5]. Measles, on the other hand, is a highly deadly disease in situations where shelter is poorly planned, people live in close proximity to each other, and immunization rates are low [4,5,6,7].

Furthermore, noncommunicable diseases (NCDs) have been seen to have a significant burden on health services post-disaster regardless of whether the event is due to natural hazards or conflict [5,8,9]. NCDs, due to prolonged treatment and the need for coordinated long-term care, pose a challenge during disaster response and recovery because of the resulting disrupted and unresponsive health system [8]. NCD treatment may either be neglected or inadequate in a disaster setting if response is poorly planned and NCDs are not considered a priority [9].

Complex emergencies are situations where livelihood and life are threatened by war, civil disturbance, and migration of people in the midst of political instability and security threats, all of which make emergency response difficult [10]. The types of injuries observed from complex emergencies may be different from disasters due to natural hazards such as typhoons and earthquakes. Violent trauma comprises one of the major direct health impacts in armed conflicts. Its effects may vary from the type and location of the conflict. In complex emergencies in Eastern Europe, violent trauma contributed to the majority of direct morbidity and mortality; however, its significance in complex emergencies in Asia and Africa was typically less due to the predominance of infectious diseases [3]. Natural hazards due to typhoons and earthquakes cause more deaths from injuries, which may be due to drowning and crush injuries, respectively [11,12,13]. For morbidity, minor injuries such as bruises, cuts, and burns played a more significant role compared to fractures in natural hazards in the Philippines in 2013 [14,15].

Surveillance for Post Extreme Emergencies and Disasters (SPEED) is a syndromic surveillance system deployed specifically after disasters and emergencies and is a joint project of the Philippine Department of Health (DOH) and the World Health Organization established in 2011 [16,17]. Disease surveillance and early warning systems have the ability to inform decision makers on possible disaster risks [18]. Multi-hazard early warning systems are integral parts of understanding disaster risk—one of the priorities under the Sendai Framework for Disaster Risk Reduction 2015–2030 [19]. Health Emergency and Disaster Risk Management (H-EDRM) is a concept that covers the intersection between disaster risk reduction management and health. It is suggested that research in this field should include needs assessments, monitoring, and evaluation and reporting systems that can measure health indicators in all phases of disasters: mitigation, preparedness, response, and recovery [20]. SPEED is an example of a reporting system in H-EDRM focused on disaster response and recovery.

Previous studies in the Philippines have looked into the health effects of natural hazards using SPEED. In the aftermath of the natural hazards in the Philippines in 2013, namely a flood, typhoon, and an earthquake, SPEED data showed that infectious diseases still dominated in morbidity; however, NCDs had a substantial burden to the health systems of the affected populations [14,15,21]. Although SPEED was also deployed in the Zamboanga armed conflict, the health effects of this complex emergency have yet to be studied using SPEED data. This study aims to describe the health consequences of the Zamboanga armed conflict on consultations for communicable diseases, injuries, and NCDs in different age groups, time periods, and health facility types using data from SPEED and their health system implications.

## 2. Materials and Methods

This study analyzed the SPEED database specifically for the Zamboanga armed conflict in 2013. SPEED was activated in Zamboanga City from 11 September 2013 to 24 March 2014, thereby beginning two days after the onset of the Zamboanga siege and spanning 196 days in total. The SPEED reports are comprised of all disease consultations for a particular day fitting the case definitions regardless of attribution to the disaster for a specific health facility, which includes village health centers, evacuation centers, hospitals, and community health centers. SPEED reports can be submitted to the system on a daily basis either manually, via Short-Messaging System (SMS), or through the internet by the designated public health nurse or data encoder [17].

Similar to previous studies done for SPEED, the 21 disease entities were divided into three groups: communicable diseases, NCDs, and injuries [14,15]. Communicable diseases included in SPEED are acute bloody diarrhea, acute flaccid paralysis, acute hemorrhagic fever, acute jaundice syndrome, acute respiratory infection (ARI), acute watery diarrhea, animal bites, conjunctivitis, fever, fever with other symptoms (FOS), suspected leptospirosis, skin disease, suspected measles, suspected meningitis, and tetanus. Fractures and wounds (including bruises and burns) make up the injury group, while noncommunicable diseases are comprised of acute asthmatic attack, acute malnutrition, high blood pressure, and known diabetes mellitus. Descriptions for each of the 21 syndromes have been mentioned by Salazar et al. in 2016 and in the SPEED Operations Manual [14,17].

The database is composed of reports with disease or syndrome counts for a particular health facility per day. There were two observed age groups: individuals under the age of five and those aged five and older. Rates were established with the denominator as either the population of the village or municipality, depending on the health facility type. Village health centers and evacuation centers used the village population, while hospitals and community health centers used the city population. The 2010 Philippine census data for villages was used for the denominator for village health center and evacuation center rates, while municipality census data was used for hospital and community health center rates. Rates were presented as per 10,000 population, similar to other studies conducted on SPEED [14,15,22].

Similar to the analysis of SPEED for typhoon Haiyan, the Bohol earthquake, and the Luzon flood in the Philippines, two time periods, less than or equal to two months and greater than two months post-disaster, were compared in this study. These time periods corresponded to disaster response and recovery, respectively [14]. To compare rates for the two disaster time periods, Poisson regression was used [23]. Consultation rates for health facility types and the mean difference between age groups (under five and five and older) were presented as mean rates with 95% confidence intervals. To illustrate total daily consultation rates among the four health facility types across the observed time period, models for each of the health facility types were derived using the multivariable fractional polynomial method [24]. Day zero for this study was pegged at the onset of the conflict.

This is a descriptive study of the SPEED database managed by the Health Emergency Management Bureau (HEMB) of the DOH. SPEED uses aggregated and anonymized data from health facilities and evacuation centers affected by emergencies and disasters. The SPEED data for the Zamboanga armed conflict was requested from the director of HEMB and the authors had exclusive access to the data.

## 3. Results

### 3.1. Health Facilities

**Figure 1:**
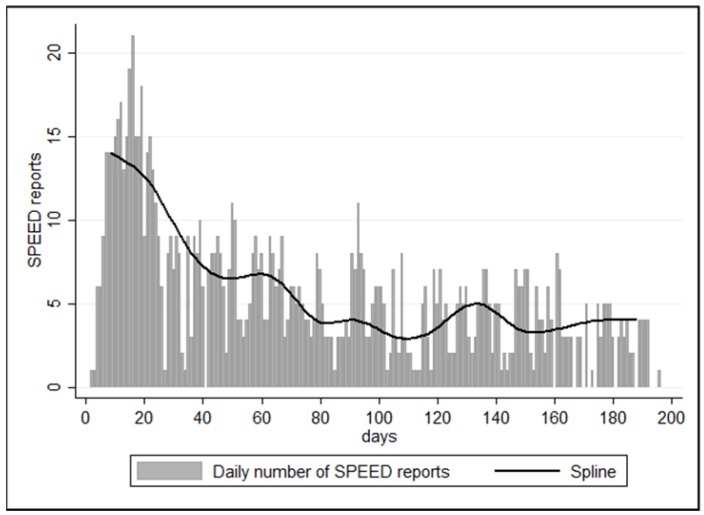
Bar graph of daily number of Surveillance in Post Extreme Emergencies and Disasters (SPEED) reports with spline.

There were 1065 SPEED reports during the Zamboanga armed conflict within a 196-day period of observation post-disaster. The first SPEED report was transmitted on the second day of the armed conflict. There was a decrease in the number of SPEED reports after the first day of reporting; however, the most notable drop happened around the 60th to 80th day, where the number of reports thereafter plateaued (see Figure 1).

Among the 49 reporting health facilities, 21 were evacuation centers, 15 were village health centers, nine were hospitals, and four were community health centers. Evacuation centers (448) had the most reports, followed by village health centers (297), hospitals (266), and community health centers (54). Comparing the total rates of consultations seen per day for the four facilities, evacuation centers (32.3 per 10,000 population) were on top, with village health centers (24.8 per 10,000 population), hospitals (0.3 per 10,000 population), and community health centers (0.2 per 10,000 population) following suit. For all health facility types, there were more daily consultations in the response period than during the recovery period (see Table 1).

**Figure 2:**
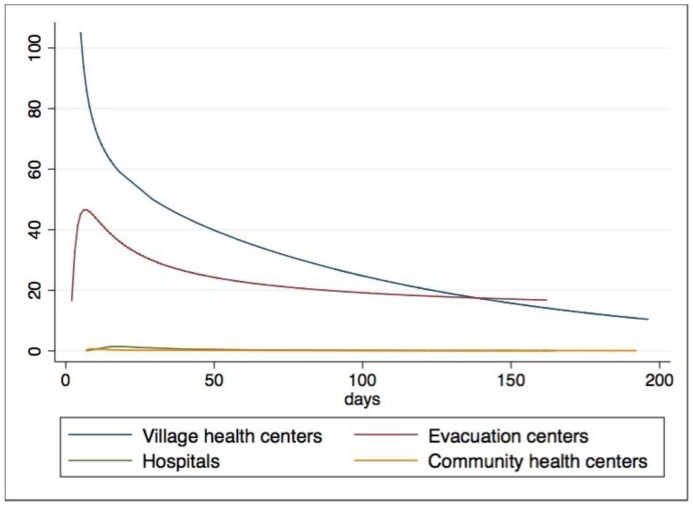
Poisson regressions for daily total consultations per 10,000 individuals across time for different health facility types.

Modeled rates for hospitals and community health centers showed little variability. Evacuation centers and village health centers differed considerably in their modeled rates. For village health centers, there was an exponential decrease in the total daily consultation rates, while evacuation centers had a delayed peak with a less profound decline (see Figure 2).

There was a notable difference between the time periods, within two months (response) and after two months (recovery), regardless of health facility type. There were similar numbers of SPEED reports for both the response (524) and recovery (541) periods. When disaggregated by health facility type, evacuation centers had more reports in the response period. The other three health facility types had more SPEED reports during the recovery period. Similar to the visualized models, village health centers had the highest difference between the rates of the two time periods. The disaster response period had significantly higher rates for all four health facilities when comparing total consultation rates (see Table 1).

### 3.2. Syndrome Rates

Communicable diseases such as acute respiratory infections (11.3 per 10,000 population), fever (3.5), acute watery diarrhea (2.3), and skin disease (1.7) had the highest syndrome rates among all 21 syndromes. Hypertension was the only disease entity that had a syndrome rate ≥1 per 10,000 population and was not communicable in nature (see Table 2). Injuries such as fractures and wounds had daily rates below 1 per 10,000 individuals, but there were more cases of wounds (1982) than of fractures (154) in terms of absolute number of cases.

The majority of the disease entities had higher rates in the disaster response period compared to the recovery period. Comparing the two disaster phases, among the 21 diseases, 13 had *p*-values less than 0.05. For communicable diseases, these were ARI, fever, acute watery diarrhea, skin disease, FOS, conjunctivitis, suspected measles, acute bloody diarrhea, and animal bites. ARI, fever, acute watery diarrhea, skin disease, acute bloody diarrhea, and animal bites had higher rates for the disaster response period compared to the recovery period. FOS, suspected measles, and acute bloody diarrhea, on the other hand, had higher rates for the disaster recovery period. The other four were wounds, bruises, and burns for injuries and high blood pressure, acute asthmatic attack, and diabetes for NCDs (See Table 2). Acute flaccid paralysis had no consults for the whole period of observation.

All the disease entities for communicable diseases had higher rates for the under-five age group, except for FOS, tetanus, and suspected meningitis. High blood pressure, diabetes mellitus, and fractures were the other diseases with higher rates for those five years and older. Of these six syndromes, the communicable diseases, FOS, tetanus, and suspected meningitis had 95% confidence intervals crossing zero.

### 3.3. Syndromes of Outbreak Potential

The DOH has a list of diseases of epidemic potential that are classified as either an immediately notifiable or a weekly notifiable disease or syndrome [25]. Among the diseases and syndromes in SPEED, acute flaccid paralysis, tetanus, animal bites (proxy for rabies), and suspected measles warrant immediate notification based on the directives of the DOH. Notifiable diseases for weekly reporting included in SPEED are the following: acute bloody diarrhea, suspected meningitis, acute watery diarrhea (which could be cholera), suspected leptospirosis, and FOS (which could be either typhoid or malaria) [25,26]. For the whole observation period, looking at the actual number of cases, there was no consult for acute flaccid paralysis, 12 for tetanus, 235 for animal bites, 60 for suspected measles, 60 for acute bloody diarrhea, 4 for suspected meningitis, 4781 for acute watery diarrhea, 31 for suspected leptospirosis, and 794 for FOS.

**Figure 3:**
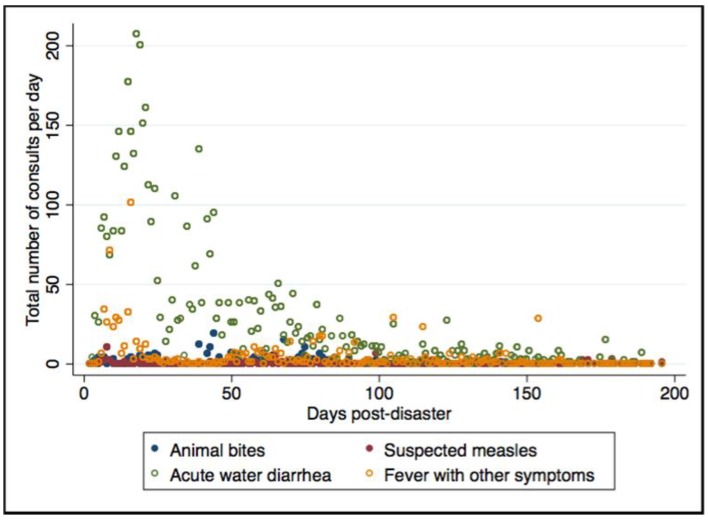
Total daily consultations of the most common immediately notifiable syndromes (animal bites and suspected measles) and the most common weekly notifiable syndromes (acute watery diarrhea and fever with other symptoms) across time among all reporting health facilities.

When visualized across time, the top immediately notifiable and weekly notifiable diseases were seen to have differing characteristics. Again, looking at the actual number of cases per day, there were more reported cases of acute watery diarrhea and FOS, which are weekly notifiable diseases, compared to animal bites and suspected measles, which are immediately notifiable diseases. Suspected measles had spikes in both the response and recovery periods, while animal bite cases had a peak in the end of the disaster response period (around the 2-month mark) and decreased thereafter. Acute watery diarrhea consistently had a higher number of cases during the disaster response period, while there were variable peaks for FOS across the observed time period (see Figure 3).

## 4. Discussion

### 4.1. Common Syndromes

In complex emergencies, communicable diseases are the usual primary care conditions [5,27]. The Zamboanga armed conflict was no exception. The top five syndromes observed can be managed in primary care settings. Similarly, in disasters in the same year (Bohol earthquake, typhoon Haiyan, and the Luzon flood), acute respiratory infections were also the most common followed by fever, acute water diarrhea, and skin disease. NCDs were also part of the top six syndromes in the form of hypertension [14,15]. However, compared to the disasters ensuing from natural hazards, wounds did not figure prominently in the disease burden in this armed conflict [14].

The top communicable disease in the Zamboanga armed conflict, ARI, reflected the yearly morbidity profile of the Zamboanga peninsula [28]. This was not the case for fever, acute watery diarrhea, and skin disease, which garnered the succeeding top numbers of consultations during the response and recovery periods post-disaster. However, the three syndromes can be associated to the environmental conditions experienced by the displaced individuals in the aftermath of the conflict, namely loss of shelter, decreased food security, and compromised access to water, sanitation, and hygiene [5,10]. The conflict displaced 80,757 individuals who were placed in 60 evacuation centers while 38,062 displaced people went to the homes of their families or relatives [1]. Those living in evacuation centers were faced with difficulties in accessing food, clean water, sanitation, and proper shelter [29]. All top four disease syndromes are communicable in nature and may be attributed to conditions in evacuation areas or temporary homes where basic services are sub-optimally provided [5,30].

NCDs represented by hypertension, the fifth most common syndrome seen in this study, are included in the top morbidities in the Zamboanga region and the Philippines in general [28]. Hypertension had been previously studied in disasters in the Philippines. In Typhoon Haiyan, hypertension was also the most common NCD and the third most common among the 21 syndromes of SPEED [15,21]. In another study done after Haiyan, hypertension was included in the top three diagnoses for adults seeking consult from mobile medical teams in 40 villages in the provinces of Samar and Leyte [31]. In 2013, hypertension was the third most common cause of morbidity in the general Philippine population, while in the Zamboanga Peninsula, the region of Zamboanga City, it was the second highest [28]. As the population is moving towards increased food availability and having sedentary work environments during normal times, people already have an increased risk of having NCDs [32]. When displaced populations who are exposed to protracted emergencies or a prolonged rehabilitation process such as in Zamboanga [29] are given packaged food with low nutritional value, have limited space for free movement, and have decreased physical activity, vulnerability to NCDs is further magnified.

Compared to other studies looking at natural hazards also using the SPEED database, the Zamboanga armed conflict had lower rates for injuries, namely wounds and fractures. In typhoon Haiyan and the Bohol earthquake, injuries resulted in rates of 1.4 and 1.2 consultations per 10,000 population, respectively. The Zamboanga armed conflict witnessed 0.7 consultations per 10,000 for injuries. The injuries seen in the typhoon and the earthquake were mostly minor in nature [14]. These injuries could be attributed to the magnitude and force of the disaster, where the affected population sustained direct injuries because of fallen structures and debris as a result of the earthquake or flying and floating debris brought about by typhoons and floods. Susceptibility to injuries is inversely related to the capacity for physical protection [33,34]. In contrast, the injuries in conflict can be attributed to gunshots or other forms of violence; however, it is hypothesized that not all people displaced by the conflict actually dealt with hazards such as gunshots, bombings, or grenades, or if they did, SPEED was not able to capture it. The consultations seen in SPEED reflected this disparity, where minor injuries were not as common in the conflict compared to natural hazards. However, these rates may also be underestimated because not all people who are injured will seek medical care or will be brought to health facilities because of several factors unique in the context of complex emergencies [35].

### 4.2. Mortality

Deaths related to violence are usually the perceived hallmark of conflicts. In Eastern Europe, trauma-related deaths predominated in the conflict in Bosnia-Herzegovina. In Asia and Africa, on the other hand, deaths due to infectious diseases and those related to malnutrition predominated [3]. In total, there were 13 deaths reported in SPEED across the observed time period ranging from day 2 to day 196. There were five deaths within the response period and eight deaths in the recovery period. There were 11 deaths reported from hospitals, while there was one death each for village health centers and community health centers. Fever was the cause of the most number of deaths among disease syndromes, with six deaths, followed by four for leptospirosis, two for hypertension, and one for wounds. The deaths reflected in SPEED, mostly communicable in nature, do not give a comprehensive picture of deaths that occurred as a consequence of the conflict.

There were differences in the deaths reported through SPEED to that of the DOH’s official mortality tally. According to the DOH, there were 268 deaths due to gunshot wounds in the Zamboanga armed conflict; however, these were not reflected in SPEED [36]. As these deaths were direct effects of the 19-day long conflict and may not have been treated in a health facility, these records could not be captured by SPEED, a health facility-dependent reporting system. In addition, as health facilities did not report daily, there were deaths that could have been missed on days in which the health facility did not send a SPEED report. Nevertheless, the reporting of deaths in the SPEED database should be further scrutinized. A time series analysis on the different systems of certifying and registering deaths after disasters, whether natural or human-induced, is suggested. Also, a delineation between direct and indirect causes of deaths will be essential. These would give a more complete picture of the impact of complex emergencies and natural disasters on mortality, such as the causes of deaths and the temporality or the relation to time post-disaster.

### 4.3. Health Facility Type

Evacuation centers had the highest number of consults per health facility per day regardless of time period post-disaster. However, during the response period to the Zamboanga armed conflict, there were more consults during the first two months post-disaster in village health centers. This may be the time when the situation had stabilized. It also shows the importance of evacuation centers as points for delivery of basic health services to the displaced population. It is hypothesized that the affected population was familiar with the village health centers, the frontline primary health care facilities, and their services, thus they sought health care in these facilities. However, as the population moved to temporary shelters, health services were delivered in the evacuation centers, explaining the large number of consultations reported from evacuation centers.

There were more SPEED reports and a higher number of consultations coming from village health centers and evacuation centers in the Zamboanga armed conflict compared to typhoon Haiyan, where SPEED was also used. In typhoon Haiyan, village health centers had the most consultations on average; however, village health centers comprised only a minority of the SPEED reports and had the highest variability. Furthermore, the distribution of consultations for health facilities across time were not discussed in publications on Haiyan using SPEED [15]. This study, on the other hand, gives a visualization of the consultations for evacuations centers and village health centers across time, showing the difference between disaster response and recovery. Reports from village health centers and evacuation centers also made up the majority of SPEED reports for this particular disaster. This study demonstrates the importance of evacuation centers and village health centers as health facilities being utilized by the displaced population to gain access to basic health services during response and recovery.

The magnitude of the disaster could have played a role in the predominance of reports from village health centers in the Zamboanga armed conflict, which was isolated to one city, compared to typhoon Haiyan, where nine regions of the Philippines were affected. The importance of community health centers in typhoon Haiyan could have reflected the need to optimize aid, since these were the main health facilities and seats of health governance of the municipalities affected [15]. Since the health system disruption of the conflict was less than typhoon Haiyan and localized within one city, relief efforts could have been distributed more to village health centers and evacuation centers.

### 4.4. Policy and Health System Implications

As part of its mandate of informing the decisions of health policy makers and providing them with early warning systems to detect possible outbreaks of conditions expected in disasters and emergencies [17], SPEED’s performance during the Zamboanga armed conflict showed that it was able to detect possible measles and cholera outbreaks. Episodes of animal bites were also recorded, which could have prompted public health measures to control stray dogs and other animals in the city. However, syndromic surveillance was shown to have limited capacity in investigating conditions that are not part of the case definitions. As disease entities are not specific and are not confirmed in the laboratory, these reported cases must be investigated further.

Event-based surveillance can feed from the real-time, location-specific reports from SPEED and lead surveillance teams to areas with unusual increases in syndrome consultations. As event-based surveillance entails rapid detection and assessment of public health events and emergencies, the team can further examine and respond to possible outbreaks identified from SPEED [37].

For SPEED to be fully utilized by public health decision makers and H-EDRM researchers, the system has to be integrated with other surveillance systems already used by the DOH. The suggestions by Foldy in 2004 to improve outcomes of surveillance systems adapted to SPEED are the following: (1) the SPEED database should be available to be mined, (2) the results from SPEED should have user-friendly visual displays or a SPEED dashboard, (3) there should be capacity for diverse technical inputs to understand complex data, (4) surveillance should be linked to response, and (5) there should be institutional capacity to confirm the diagnosis, both clinical and laboratory [38]. This entails coordination among different bureaus within the DOH. SPEED is under the Health Emergency Management Bureau, while the other surveillance systems are under the Epidemiology Bureau of the DOH. Moreover, it was observed that health facilities were not consistent in sending daily SPEED reports. The gaps in the reporting in SPEED, whether in terms of morbidity and mortality, shows the need for further validation through different data sources within and outside government to get the best picture of the complex emergency or disaster. It is also prudent to assess the reporting system for deaths post-disasters in health facilities and investigate the process of certification and reporting. These can provide information on the mechanisms of death, classification, and possible public health interventions.

Since disasters from natural hazards and complex emergencies amplify health systems’ weaknesses and needs [10,14], other health services should be monitored aside from infectious diseases, non-communicable diseases, and injuries. Mental health services during and after conflicts are essential during disaster response and recovery. In a humanitarian setting, mental health conditions could be preexisting, trauma-induced, or triggered by failures in basic services during response and recovery [39,40]. On the other hand, women still needed to access maternal health services during the on-going conflict in Zamboanga. Consultations for antenatal, delivery, and postnatal care were seen in evacuation centers and in health facilities [41]. Aside from emergency obstetric care, the minimum initial service package in humanitarian emergencies also includes other interventions under reproductive health, such as those for sexual violence, contraception, and HIV [42]. When it was first developed in 2011, SPEED did not include syndromes or diseases pertaining to mental health or maternal health. The inclusion of mental health and reproductive health in SPEED can be further studied by the DOH to be able to capture important concerns that can guide provision of more specific health services.

### 4.5. Limitations

The SPEED database is dependent on the use of the health workforce who may be preoccupied and overworked during times of emergencies and disasters. The use of the system and its activation is dependent on the decisions of either the local chief executive, health officer, or the DOH. This in turn affects the allocated human resources and logistics deployed to support SPEED’s operational requirement [17]. The required continued monitoring and governance by health officials coupled with other external stressors in disaster response and recovery could have led to the inconsistencies in the reporting of health facilities in the aftermath of the complex emergency.

The data quality of SPEED needs to be further evaluated even though SPEED results are initially validated by HEMB [14]. As a secondary data source without confirmed diagnoses, results from SPEED can only be used for descriptive studies and not for confirming the need for declaring outbreaks [17]. Furthermore, as SPEED is housed in HEMB and isolated from other disease surveillance systems, coordination among DOH bureaus needs to be improved in order to have an integrated disease surveillance and response system both in disasters and in normal times.

## 5. Conclusions

The SPEED data for the Zamboanga armed conflict showed that primary care conditions such as common infections and NCDs were the most prevalent reasons for consultations. Evacuation centers had the highest rates and the highest number of SPEED reports, thus highlighting the importance of the health facilities nearest to the population affected. Common infectious diseases, such as ARI, fever, watery diarrhea, and skin disease, may be associated with environmental conditions of displaced populations in contexts wherein essential health services are lacking or inadequate. Hypertension, on the other hand, is a common condition needing chronic and long-term care. These forms of considerations validate the emphasis that must be placed on primary health care in the immediate aftermath of disasters, whether natural or human-induced. Lastly, SPEED was able to assist decision makers on identifying possible outbreaks, which were averted; however, it did not pick up a high number of injuries and deaths due to violence expected from complex emergencies. SPEED is also in isolation from other reporting systems. It is suggested that linkages between SPEED and other surveillance systems, such as event-based surveillance and indicator-based surveillance and other reporting systems used especially for trauma and deaths, be strengthened to better illustrate the public health impacts of complex emergencies. Coordination within the DOH needs to be improved in order to have a truly integrated disease surveillance system.

## Figures and Tables

**Table 1:** Total consultation rates per 10,000 individuals for health facility types comparing time post-disaster.

Health Facility Type	Mean Number of Consultations per Day (95% Confidence Intervals) (*n*)	≤2 Months (Response) (*n*)	>2 Months (Recovery) (*n*)	Difference between ≤2 Months and >2 Months (*p*-value)
Evacuation center	32.3 (27.9–36.7) (*n* = 448)	34.3 (*n* = 373)	22.4 (*n* = 75)	11.9 (<0.01)
Village health center	28.5 (24.5–32.5) (*n* = 297)	65.5 (*n* = 70)	17.1 (*n* = 227)	48.4 (<0.01)
Hospital	0.3 (0.2–0.3) (*n* = 266)	0.5 (*n* = 68)	0.2 (*n* = 198)	0.3 (<0.01)
Community health center	0.2 (0.1–0.2) (*n* = 54)	0.4 (*n* = 13)	0.1 (*n* = 41)	0.3 (0.03)
Regardless of facility type	21.6 (19.3–23.9) (*n* = 1065)	33.2 (*n* = 524)	10.3 (*n* = 541)	22.9 (<0.01)

**Table 2:** Top syndrome rates per 10,000 individuals separated by time post-disaster and by age.

Syndrome	Total *n* = 1065	≤2 Months (Response) *n* = 524	>2 Months (Recovery) *n* = 541	Difference between ≤2 Months and >2 Months (*p*-value)	<5 Years of Age	≥5 Years of Age	Difference between <5 Years and ≥5 Years (95% Confidence Interval)
Communicable diseases							
Acute respiratory infection (ARI)	11.3	18.0	4.8	13.2 (<0.01)	41.8	7.2	34.7 (30.7–38.6)
Fever	3.5	4.9	2.2	2.7 (<0.01)	14.0	2.1	11.8 (10.1–13.6)
Acute watery diarrhea	2.3	3.8	0.9	2.9 (<0.01)	8.5	1.5	7.0 (5.9–8.1)
Skin disease	1.7	2.4	1	1.4 (<0.01)	5.7	1.2	4.5 (3.7–5.3)
Fever with other symptoms (FOS)	0.3	<0.1	0.1	0.5 (<0.01)	*0.3*	*0.3*	<0.1 (−0.1–0.2)
Communicable disease total	19.4	29.9	9.3	20.6 (<0.01)	71.1	12.5	58.6 (52.0–65.2)
Injuries							
Open wounds and bruises/burns	0.7	0.9	0.4	0.4 (<0.01)	0.9	0.6	0.3 (0.1–0.5)
Injury total	0.7	0.9	0.4	0.4 (<0.01)	0.9	0.6	0.3 (0.1–0.5)
Non-communicable diseases (NCDs)							
High blood pressure	1.0	1.8	0.3	1.4 (<0.01)	<0.1	1.2	1.2 (1.0–1.4)
Acute asthmatic attack	0.4	0.6	0.2	0.4 (<0.01)	1.1	0.3	0.8 (0.6–1.0)
NCD total	1.5	2.4	0.6	1.8 (<0.01)	1.4	1.5	0.1 (−0.3–0.5)
